# Bilateral hip exoskeleton assistance enables faster walking in individuals with chronic stroke-related gait impairments

**DOI:** 10.1038/s41598-025-86343-x

**Published:** 2025-01-15

**Authors:** Chiara Livolsi, Roberto Conti, Tommaso Ciapetti, Eleonora Guanziroli, Thor Fridriksson, Ásgeir Alexandersson, Emilio Trigili, Francesco Giovacchini, Raffaele Molino Lova, Alberto Esquenazi, Franco Molteni, Simona Crea, Nicola Vitiello

**Affiliations:** 1 The BioRobotics Institute, Scuola Superiore Sant’Anna, Pontedera, Pisa, Italy; 2 Department of Excellence in Robotics & AI, Scuola Superiore Sant’Anna, Pisa, Italy; 3 IUVO S.r.l, Pontedera, Pisa, Italy; 4 IRCCS Fondazione Don Carlo Gnocchi ONLUS, Florence, Italy; 5 Villa Beretta Rehabilitation Center, Valduce Hospital, Costa Masnaga, Lecco, Italy; 6 Össur, Reykjavík, Iceland; 7 Department of PM&R, Jefferson Moss-Magee Rehabilitation, Elkins Park, PA USA

**Keywords:** Biotechnology, Health care

## Abstract

Millions of individuals surviving a stroke have lifelong gait impairments that reduce their personal independence and quality of life. Reduced walking speed is one of the major problems limiting community mobility and reintegration. Previous studies have shown positive effect of robot-assisted gait training utilizing hip exoskeletons for individuals with gait impairments due to a stroke, leading to increased walking speed in post-treatment compared to pre-treatment assessments. However, no evidence emerged of a significant increasing in walking speed attributable to device usage compared to walking without the device. In this pilot investigation, we observed that hip flexion/extension assistance delivered by a portable bilateral powered hip exoskeleton increased overground self-selected walking speed by 20.2 ± 5.0% on average among six chronic post-stroke survivors. When comparing walking with and without the hip exoskeleton within the same experimental session, the observed speed increment resulted in statistically and clinically meaningful improvement (0.14 ± 0.03 m/s > minimal clinically important difference, *p* = 0.015). The increased walking speed was the result of a higher self-selected cadence and longer step length both on the paretic and nonparetic limbs. By facilitating gait, a bilateral hip exoskeleton could be a viable technology for extending locomotor mobility and facilitating gait training of individuals affected by post-stroke hemiparesis.

**Supplementary Information:**

The online version contains supplementary material available at 10.1038/s41598-025-86343-x.

## Background

With 12.2 million new episodes occurring every year^1^, stroke is a prevalent cause of morbidity and long-term disability worldwide^2^. Among stroke-induced dysfunctions, gait impairments are one of the major factors impacting the quality of life and personal independence. Accordingly, gait recovery is among the most desirable goals of stroke rehabilitation. However, even after completing conventional rehabilitation therapies most stroke survivors experience persistent gait impairments that limits their activities of daily living^3^. Hemiparetic gait is characterized by unilateral muscle weakness and/or spasticity, joint motion deviation, compensatory movements, and impaired interlimb coordination, which make walking slower, more demanding and fatiguing.

Self-selected speed stands out as a key performance measure of walking, as it reflects complex interplay of multiple body structures and functions such as lower extremity strength, proactive and reactive postural control, interlimb coordination, aerobic capacity, proprioception, and vision^4^. The comfortable walking speed is strongly associated to functional independence in real-word scenarios. Perry et al. showed that gait speed can discriminate among different levels of functional walking categories and serve as a predictor of home and community ambulation following a stroke^5^. According to a recent analysis, a comfortable gait speed of 0.49 m/s discriminates between home and limited community ambulators and a comfortable walking speed higher than 0.93 m/s enables unrestricted community ambulation, which only 12% of individuals achieve after a stroke^6^. Reduced walking speed is one of the major limitations to community mobility and reintegration, as it limits essential tasks likely safely crossing streets within the green-yellow phase of traffic lights^7^. Therefore, enhancing walking speed is of paramount importance for restoring independence and mobility among stroke survivors^8^.

Wearable robotic devices can play a key role in gait rehabilitation in overground ecological conditions. Multi-joint lower limb exoskeletons have shown potential in enabling individuals with severe neurological gait impairments to walk again^8^, whereas single-joint powered exoskeletons, targeting the hip, knee, or ankle, can represent a compact, tailored yet effective solution for augmenting personal mobility in individuals with mild to-moderate gait impairments. However, current clinical evidence demonstrating the feasibility of increasing walking speed through single-joint assistance in individuals who retain some degree of walking capacity, such as the majority of stroke survivors (up to 80%)^9^, is still limited.

In gait biomechanics, focusing on the roles of individual joints, the ankle and hip contribute significantly to generating positive mechanical work^10^,with the hip being less efficient than the ankle due to the proximal-distal gradient of the muscle-tendon architecture^11^. Previous studies revealed that limited walking speed in post-stroke individuals is mostly due to reduced capacity of power generation at the paretic hip and ankle^12,13^. Specifically, the hip power has been recognized as a key determinant of walking capacity and serves as a predictor of maximal walking speed^12–15^.

Previous studies have investigated the use of hip exoskeletons for gait training in individuals with chronic stroke, revealing a positive effect on speed, following intensive training (i.e., four weeks, three sessions/week)^16–19^or even short training programs (i.e., five days)^20^. These studies have shown increased walking speed in post-treatment compared to pre-treatment assessment. However, the effects reported in those studies may have been confounded by gait training itself, and the specific role of the exoskeleton in increasing walking speed has not been thoroughly investigated.

Evaluating gait speed while walking with versus without the device may be relevant to understanding whether, and to what extent, exoskeleton assistance could have enhanced the training program and, more generally, whether the device could lead to better gait performance when used as assistive technology in daily activities.

A few previous studies have preliminarily investigated the assistive effect of hip exoskeleton assistance on walking speed in post-stroke survivors. A study demonstrated a clinically significant increase in walking speed in a single participant^21^, other three studies observed changes that were not clinically meaningful or changes that could be partly attributed to motor learning^22–24^.

In such a framework, this study explores whether using a hip exoskeleton can lead to increased walking speed in individuals with chronic stroke in comparison to walking without the device within a single experimental session. Effects on gait biomechanics were evaluated as changes in overground walking speed and spatiotemporal parameters.

## Results

We conducted experimental tests with six individuals in the chronic post-stroke phase walking with and without an Active Pelvis Orthosis (APO). The APO is a bilateral powered hip exoskeleton designed to assist with hip flexion and extension during gait by providing adaptive torques at the hip joints, seamlessly accommodating natural gait variations^25^. Study participants were recruited among individuals capable of independent gait, with or without tool such as cane or ankle foot orthosis, and exhibiting mild-to-moderate gait impairments i.e., Functional Ambulation Classification ≥ 4 and Medical Research Council muscle strength score in the range (2,4], (Supplementary Table I). Participants walked overground on a 23-m walkway and on a treadmill at their self-selected speed. The APO provided assistive torque in the sagittal plane in the hip flexion and/or extension direction (patent application nr. WO2022137031)^26^. Self-selected walking speed was assessed through 10-meter walk tests (10mWT) overground. Spatiotemporal parameters were measured using an instrumented walkway during overground walking, while gait kinematics was obtained using a motion capture system during treadmill walking. Before these assessments, each subject participated in three experimental sessions, the first for the enrollment, the second for the tuning of the hip assistance, and the third for the familiarization with the hip exoskeleton assistance (Experimental protocol).

### Overground test: gait speed and spatiotemporal parameters

The self-selected comfortable walking speed was measured at the steady state during 10mWT performed in a 23-m walkway.

For each participant, walking speed was assessed under three different walking conditions: (i) walking without the APO (NoAPO), (ii) walking with the APO without assistance (transparent mode, TM), and (iii) walking with the APO with assistance (assistive mode, AM). Within the session, the NoAPO condition was executed at the baseline (NoAPO_baseline_), and at the end of the session (NoAPO_final_), i.e., before and after tests with the APO; and the order of APO conditions (TM, AM) was randomized across participants to avoid potential order bias.

**Fig. 1:**
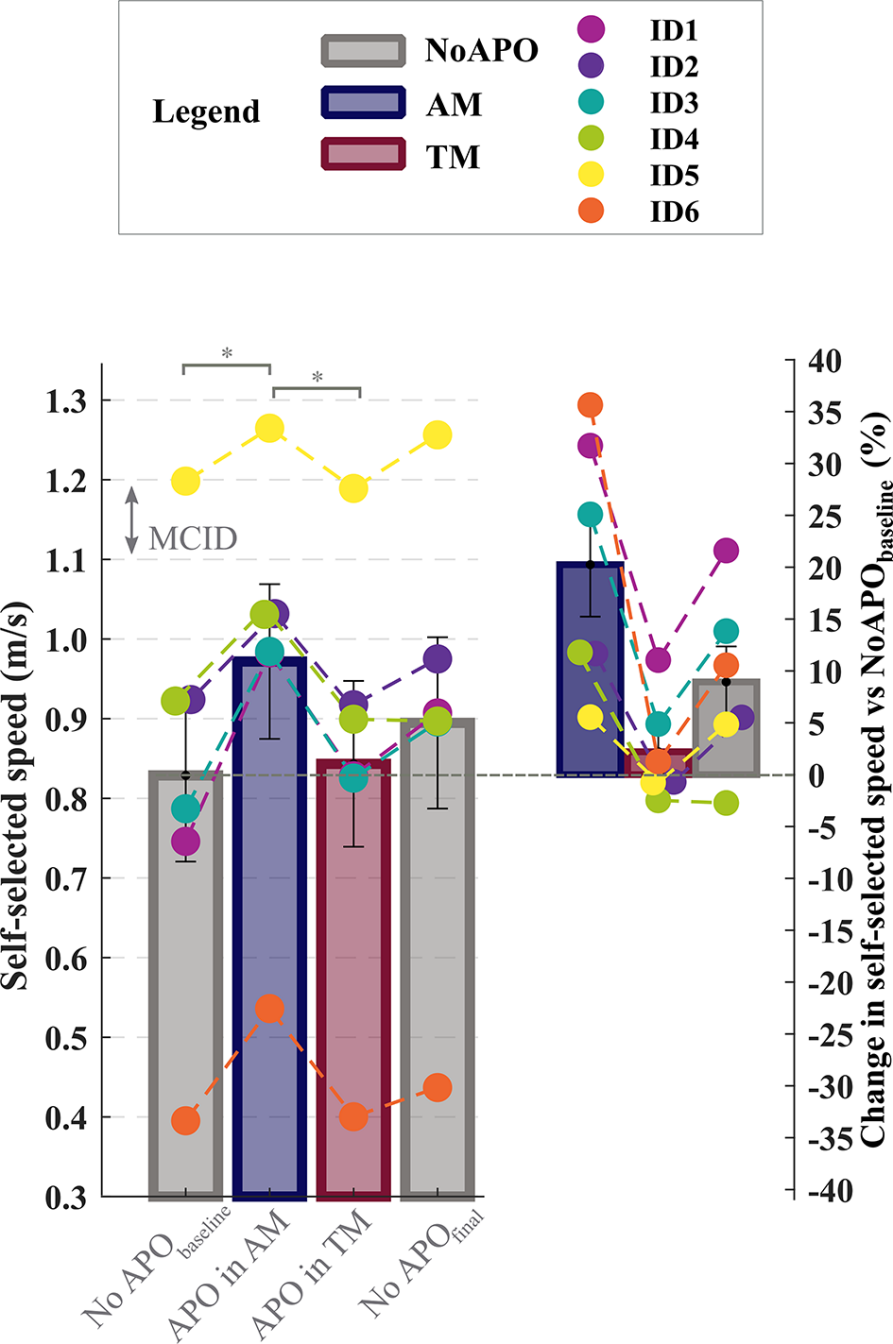
Self-selected walking speed measured during 10mWTs with and without the APO. On the left, colored bars represent the self-selected comfortable walking speed (m/s) measured during the 10mWTs in the following experimental conditions: (i) without the APO at the beginning and at the end of the session (NoAPO_baseline_ and NoAPO_final_, grey), (ii) with the APO in assistive mode (AM, blue), (iii) with the APO in transparent mode (TM, bordeaux). The minimal clinically important difference (MCID) is indicated with dashed horizontal lines. On the right, colored bars represent the percentage change in the self-selected walking speed with respect to NoAPO_baseline_. Aggregated data across participants are reported as means and standard error means. Colored dots represent individual study participants. The square brackets with the asterix indicate the statistically significant differences when comparing APO in AM versus NoAPO_baseline_ and APO in TM (post-hoc test *p* < 0.05).

The self-selected speed was 0.83 ± 0.11 m/s (mean ± standard error mean, s.e.m.) without the APO (NoAPO_baseline_), 0.97 ± 0.10 m/s with the APO in AM, 0.84 ± 0.10 m/s with the APO in TM, and 0.89 ± 0.11 m/s without the APO at the end of the session (NoAPO_final_), (Fig. 1). The APO assistance induced a statistically significant (*p*< 0.05) and clinically meaningful increment. in participants’ self-selected walking speed., Specifically, the observed gait speed increment exceeded the 0.10 m/s minimal clinically important difference (MCID)^6^ with a 20.2 ± 5.0% (0.14 ± 0.03 m/s, *p* = 0.015) compared to the baseline without the device (NoAPO_baseline_), and with a 15.0 ± 3.8% (0.13 ± 0.01 m/s, *p* < 0.001) compared to walking with the APO in TM. No significant changes in walking speed were observed walking with the APO in TM compared to walking without it. Similarly, the comparison between NoAPO_final_ and NoAPO_baseline_, showed an 8.9 ± 3.4% increase in walking speed (0.06 ± 0.03 m/s, *p* > 0.05), but this change did not reach statistical significance.

**Fig. 2:**
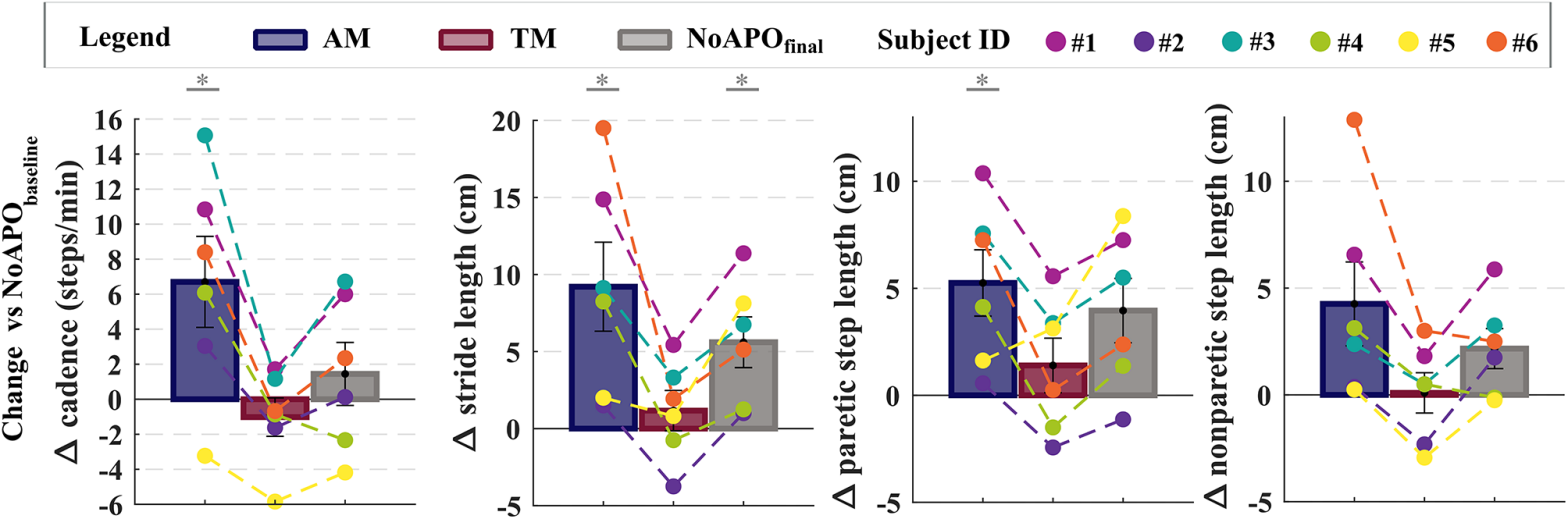
Changes in spatiotemporal parameters measured during the 10mWTs with and without the APO. Colored bars represent the variation in the spatiotemporal parameters in different walking conditions, compared to walking without the APO at the beginning of the session (NoAPO_baseline_). Walking conditions are the following: walking with the APO providing assistance (AM, blue), with the APO in transparent mode (TM, bordeaux) and without APO at the end of the sesison (NoAPO_final_, grey). From left to right, spatiotemporal parameters are cadence (steps/min), stride length (cm), paretic step length (cm), nonparetic step length (cm). Aggregated data are reported as means and standard errors. Colored dots represent individual study participants. The asterix indicate the statistically significant differences when comparing to NoAPO_baseline_ (post-hoc test *p* < 0.05).

We computed spatiotemporal parameters during the 10mWTs by considering steps in the five meters in the center of the walkway. The cadence, the stride length, and the paretic step length significantly increased with the APO in AM compared to the APO in TM, and to the walking conditions without APO (Fig. 2). Specifically, the cadence significantly increased when walking with APO in AM (97.1 ± 6.5 steps/min) by 6.7 ± 2.6 steps/min (*p* = 0.048) compared to the NoAPO_baseline_ (90.4 ± 6.9 steps/min) and 7.7 ± 3.9 steps/min (*p* = 0.004) compared to APO in TM (89.4 ± 6.7 steps/min). The cadence increased in the NoAPO_final_ condition (91.8 ± 6.6 steps/min) by 2.5 ± 0.9 steps/min compared to APO in TM (*p* = 0.056). Concurrently, the stride length significantly increased compared to the NoAPO_baseline_ (110.0 ± 10.1 cm) by 9.2 ± 2.9 cm (*p* = 0.024) in AM and 5.6 ± 1.6 cm (*p* = 0.046) in the NoAPO_final_. The increase in step length observed in AM compared to NoAPO_baseline_was higher than the minimal detectable change (MDC) both on the paretic and nonparetic side^27^ (i.e., 5.3 ± 1.5 cm > 2.6 cm on the paretic side and 4.2 ± 1.5 cm > 2.1 cm on non-paretic side). Statistically, the paretic step length significantly increased by 5.3 ± 1.5 cm (*p* = 0.019) walking with the APO in AM to NoAPO_baseline_, and 3.9 ± 1.2 (*p* = 0.024) cm compared to TM. In the condition NoAPO_final,_ the paretic step length also increased by 4.0 ± 1.5 cm (*p* = 0.006) compared to NoAPO_baseline_. The nonparetic step length increased significantly, by 4.2 ± 1.2 cm (*p* = 0.018), only walking with the APO in AM compared to TM. No other significant changes in nonparetic step length were observed across the tested conditions. Also, we analyzed temporal and spatial gait symmetry and did not find significant differences with and without the APO (*p* > 0.05, Supplementary Table II).

## Overground test: hip kinematics and kinetics

**Fig. 3:**
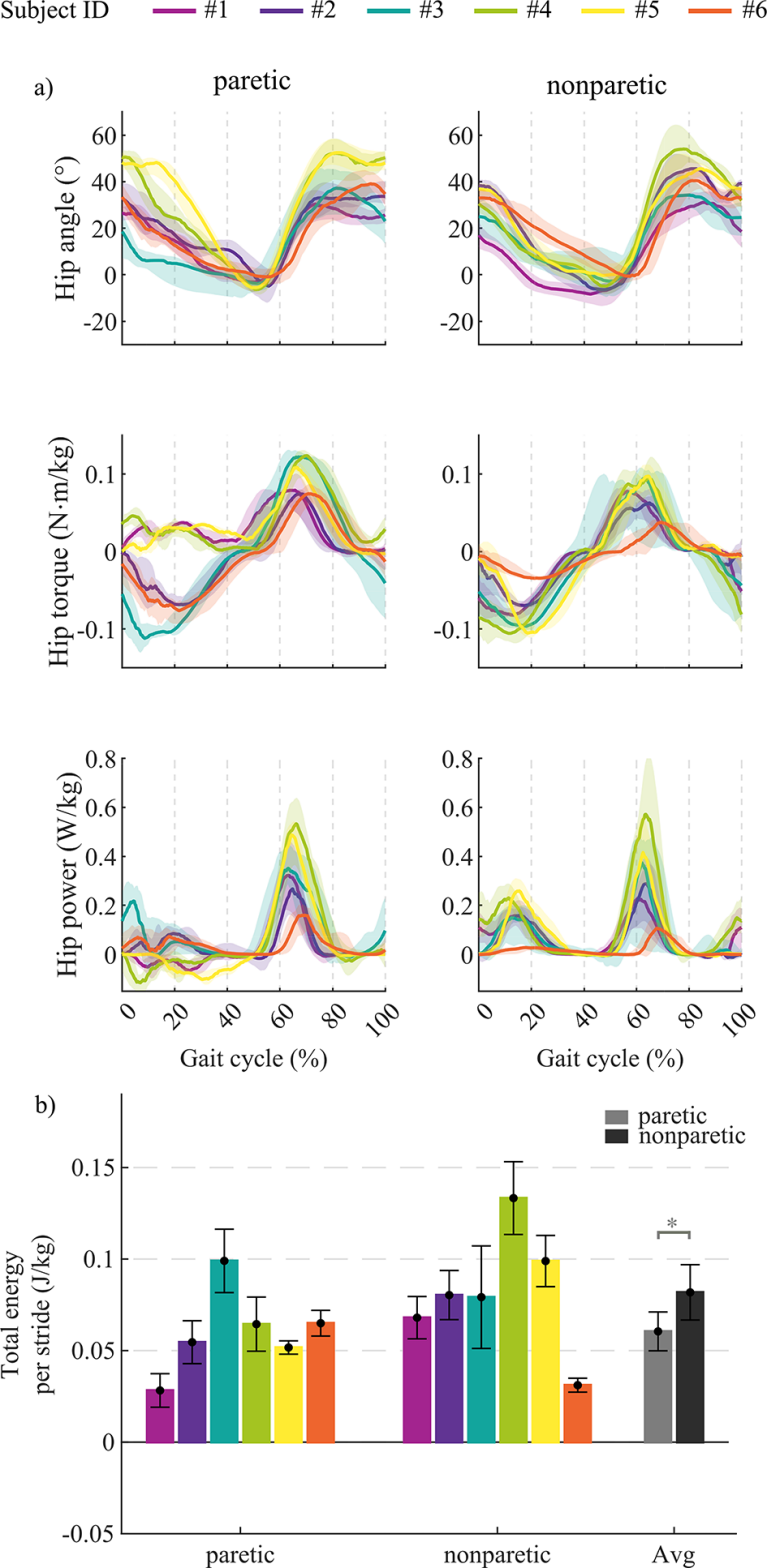
Hip angle, torque, and power measured during the 10mWTs with the APO in assistive mode. (a) The hip angle measured by APO encoders, the APO hip torque and power normalized to body mass. The angle, torque, and power profiles are shown for each participant; each profile is depicted by the average (solid line) and standard deviation (shadow area) over all the strides of the 10mWT in AM. (b) APO energy injected during the entire stride duration, normalized to body mass. All bar plots represent means and standard deviations computed over all the strides of the 10mWTs in AM for each participant. On the right, the averaged values across participants (Avg) for paretic (grey) and nonparetic side (black), that resulted statistically different (paired-ttest, *p* < 0.05).

During the overground walking test, bilateral hip angles and provided torques were measured using the APO onboard hip encoders. In the AM condition, the hip kinematics and kinetics varied noticeably between participants, indeed, the hip range of motion (ROM) was in the range [33.5, 58.5] ° and [36.9, 58.9] ° respectively on the paretic and nonparetic sides (Fig. 3). On the paretic side, the peak of the flexion torque provided by the APO during the swing phase ranged between 0.07 N·m/kg and 0.12 N·m/kg, corresponding to a positive power of 0.36 ± 0.06 W/kg. Throughout the stance phase, the torque provided by the APO ranged from − 0.11 to 0.05 N·m/kg. This range corresponds to a peak of mechanical power within the range of [−0.12, 0.22] W/kg.

On the nonparetic side, the APO provided an average flexion torque of 0.08 ± 0.01 N·m/kg and extension torque of 0.08 ± 0.01 N·m/kg respectively during the swing and stance gait phase, corresponding to a positive power of 0.16 ± 0.08 W/kg and 0.33 ± 0.16 W/kg, respectively.

From the energetic standpoint, the total energy per stride provided by the APO was in the range [0.03, 0.1] J/kg on the paretic side and in the range [0.03, 0.13] J/kg on the paretic side (Fig. 3b). By averaging across participants, the total energy per stride on the paretic and nonparetic side resulted statistically different (paired-ttest, *p* < 0.05).

## Treadmill test: gait kinematics

**Fig. 4:**
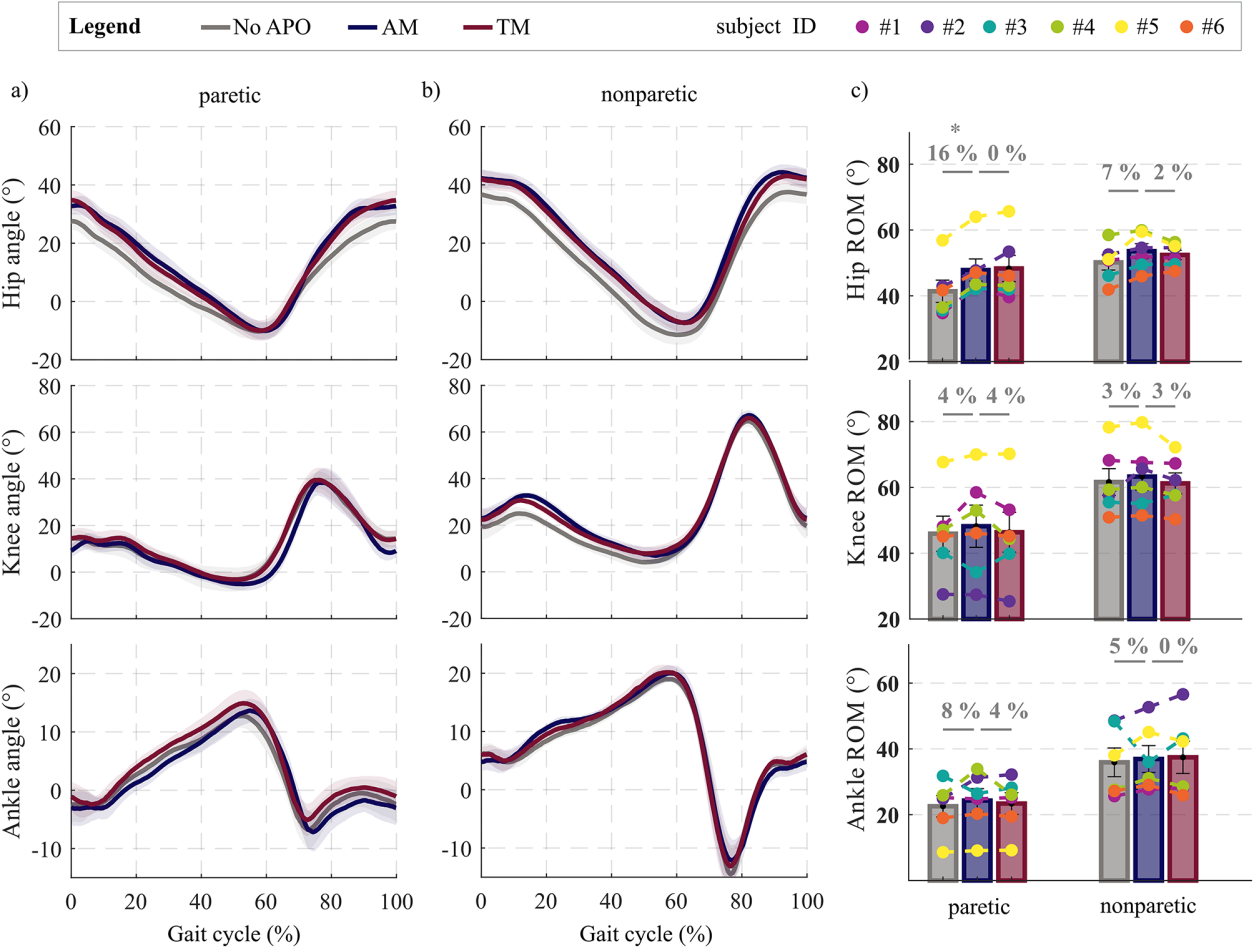
Lower-limb kinematics during treadmill walking with and without the APO. a, b) Hip, knee and ankle angle profiles of the paretic (a) and nonparetic limbs (b) during treadmill walking in the following conditions: (i) walking without the APO (NoAPO, grey), (ii) walking with the APO in assistive mode (AM, blue), (iii) walking with the APO in transparent mode (TM, bordeaux). Aggregated profiles across participants are shown as mean (solid line) ± s.e.m. (shadow area). c) Sagittal range of motion (ROM) of the hip, knee, and ankle joints of the paretic (left) and nonparetic (right) side. Aggregated data are reported in bar plots as mean ± s.e.m (*n* = 6) for the different walking conditions (NoAPO, AM, TM). Colored dots represent individual study participants. The percentage changes in AM with respect to NoAPO and TM are indicated above the bar plots. The asterix indicate the statistically significant differences when comparing APO in AM versus NoAPO_baseline_ (post-hoc test *p* < 0.05).

All participants walked on a treadmill without and with the APO, in both TM and AM, at their self-selected speed. The self-selected speed on the treadmill was selected by each subject with the APO in AM and the same speed was maintained for tests without the APO and with the APO in TM. On average, participants walked slower than overground. We computed the average hip, knee, and ankle angle profiles across all participants during 1 min of treadmill walking in each condition. The ROM of the paretic hip joint was significantly larger (16%, *p* = 0.011) with the APO in AM than in NoAPO (Fig. 4). Specifically, the average ROM of the paretic hip was 41.4 ± 3.4° in the NoAPO condition, and 47.8 ± 3.4° with APO in AM. No significant differences were observed in the ROM of other joints (*p* > 0.05, Fig. 4).

## Discussion

Stroke causes gait deficits reducing functional ambulation in the community^28^. In this pilot investigation, the bilateral hip assistance provided by the APO led to increased overground self-selected walking speed of six individuals with hemiparetic gait due to a stroke, compared to walking without the APO. The observed increment was on average by 20.2 ± 5.0% (namely, 0.14 ± 0.03 m/s) and was statistically and clinically meaningful (i.e., *p*< 0.05 and increment > 0.10 m/s minimal clinically important difference, MCID)^6^.

Recently, Young and colleagues examined the effect of a hip exoskeleton on gait speed, fall risk, gait symmetry and energy expenditure in a group of nine stroke survivors. However, no differences between the exoskeleton and the baseline conditions were observed for any outcomes^23^. Pan and colleagues investigated the effects of a hip exoskeleton on five post-stroke subjects and reported a change of the self-selected comfortable speed of −0.7% and + 17.6%, respectively comparing TM and AM with the baseline without the exoskeleton^22^. The increment in the gait speed reported in the cited study is similar to the one reported in the present work, even though some methodological differences may call for more in-depth considerations. In the present study, the walking condition without and with the exoskeleton in TM and AM were measured within the same experimental session. Instead, in the abovementioned study, the walking speed without the exoskeleton (the so-called “baseline” condition) was measured in an experimental session prior to the assessment session with the exoskeleton, thus part of the reported increment could be attributable to various factors, such as practicing the walking task, the familiarization/accommodation with the testing setup and day-to-day variability. Indeed, participants performed a familiarization session between the baseline session and the test session, which likely contributed to making participants feel confident with the walking test, set-up, and environment and therefore to walking at higher pace across days, even without the exoskeleton. This hypothesis is consistent with the findings of our previous study^20^where we observed an increment of 18.8% in the gait speed measured without the device at the test session (day#3) compared to the baseline (day#1) with a familiarization session in the middle (day#2). In addition to practicing effects, the day-to-day variability of walking performance in post-stroke individuals can be as large as 50% of the mean^29^, thus, any day-to-day evaluation may reflect high variability. Considering that practicing effects could be relevant also within a single experimental session, due to the multiple repetitions of the task, in the present study the NoAPO condition was performed twice, before and after the APO conditions, making it possible to quantify the practicing effects along the session. Indeed, an average increment of + 8.9 ± 3.4% in gait speed has been measured in the NoAPO_final_ versus the NoAPO_baseline_, that could be attributed both to practicing and post-removal retention effects. The so called “retention effect” is an important aspect to consider when evaluating assistive devices. This is namely the capacity to elicit modifications in the subject’s behavior that could be retained even when the device is taken off. In this study, the gain measured in the NoAPO_final_ versus the NoAPO_baseline_, even if not statistically significant, may suggest a positive trend that could indicate that subjects may have started modifying their internal gait scheme to cooperate with the device and that mechanical and neural adaptation occurred^30^. Future clinical investigations should aim to elucidate the distinctive signatures of motor and neural adaptation attributable to the two factors: (i) robotic hip assistance and (ii) walking task practising.

Two studies compared the gait performance of stroke survivors walking with and without a hip exoskeleton within the same session. The study by Firouzi and colleagues^24^, reported a small increment of gait speed of 5.7% on average in five post-stroke individuals walking with the Stride Management Assist (SMA) hip exoskeleton in AM compared to the baseline without the device during the same test session, however, this average increase of 0.05 m/s was not clinically meaningful (i.e., < the small MCID 0.06 m/s)^31^. The second study compared the gait performance of one single stroke survivor walking with and without a unilateral hip exoskeleton within the same session and reported a clinically relevant increase in the walking speed of 29.6% (i.e., 0.16 m/s > MCID 0.10 m/s)^21^. Hence, the present study confirms the preliminary finding with a larger group of subjects and reports a clinically meaningful speed increment between 0.07 m/s and 0.24 m/s, i.e., higher than the small MCID (0.06 m/s) for one subject (ID5), and higher than the substantial MCID (0.10 m/s)^31^ for the other five subjects (ID1-4,6). This finding shows the feasibility to augment walking speed using a powered hip exoskeleton in stroke survivors with mild impairments.

Analyzing the speed gains relative to participants’ initial walking performance reveals that percentage improvements in walking speed using the APO in AM was larger for participants with lower baseline mobility (Supplementary Fig. 1). The baseline walking speed varied noticeably among study participants, ranging from 0.4 m/s to 1.2 m/s, with the larger increases in walking speed occurring in participants with low walking speed, up to an increment of + 36% with the subject whose initial self-selected walking speed was 0.4 m/s. This result may be explained by the fact that hemiparetic gait is typically characterized by ineffective recruitment of hip flexor muscles of the paretic side^12^, which tend to be more pronounced in individuals with lower functional mobility. For example, the participant ID6 who was the oldest and one with the lowest baseline walking speed, achieved a substantial increase of 0.16 m/s. Indeed, hip strength is among the major determinants of comfortable and fast walking speed in physiological gait^15,32^, therefore the hip flexion torque provided by the APO may have played a more important role in subjects with very limited hip strength. Furthermore, the participant ID5, the youngest in the study, exhibited a baseline walking speed that was notably higher than other participants, with a comparatively smaller increase in walking speed of 0.07 m/s. This limited improvement may be attributed to the participant’s already optimized walking mechanics for fast speed, which may limit the potential for further gains when assisted by the device. This observation suggests that older individuals or those with greater impairments might derive more substantial benefits from exoskeleton assistance, likely due to their existing limitations in mobility. This aligns with findings of a previous study evaluating differences in outcomes between post stroke individuals categorized as limited community ambulator (comfortable speeds below 0.93 m/s) and full community ambulators (comfortable walking speeds above 0.93 m/s) when using an ankle exoskeleton^33^. If these trends are confirmed in larger studies, it is likely that higher improvements may happen with subjects with even lower functional walking levels than participants recruited in this work, i.e., with a cohort of household ambulators. Variability in exoskeleton response can be influenced by multiple factors, including age-related physiological changes, the severity of the impairment and individual motivation and familiarity with the assistive devices. By considering these factors, further studies are necessary to better understand variations in gait performance and to support the development of personalized rehabilitation approaches.

In this preliminary investigation, five out of six participants increased their functional walking category according to the classification proposed by Fulk and colleagues^6^; in particular, one subject (ID6) increased the gait speed from 0.4 m/s to 0.53 m/s, therefore passing from household ambulator to limited community ambulator, and four subjects (ID1-4), initially classified as limited community ambulators, reached with the APO a gait speed typical of community ambulators^6^(namely, higher than 0.93 m/s), approaching or surpassing the value of 1 m/s that is also considered an indicator of reduced fall risk among community-dwelling people^34^. In the case this functionality in enabling individuals to achieve a higher level of mobility will be confirmed in a large clinical study the impact could be relevant.

From a biomechanics perspective, the increase in gait speed observed in overground walking was the result of a higher cadence and longer steps both on the paretic and nonparetic sides, indicating that the increased gait speed was accompanied by improved gait quality as longer step length is typically associated with higher gait stability and reduced risk of fall^35^. In addition, all study participants were able to walk on the treadmill with the proposed hip assistance without noticeable changes in gait kinematics. Overall, a greater benefit induced by the hip assistance was observed in overground walking performance as compared to kinematic modifications during treadmill walking. However, in evaluating this difference, it should be noted that treadmill walking is intrinsically different as it does not allow users to increase the step length and cadence at the same time, therefore different adaptation mechanisms should occur to modify the gait scheme that may require a longer training period. Considering the short duration of the familiarization on the treadmill (5 min), compared to the familiarization time overground (40 min), the absence of kinematic differences in the AM and NoAPO conditions observed on the treadmill may be not conclusive and may require dedicated investigations.

To evaluate whether wearing the passive structure of the APO negatively affected hemiparetic gait, the walking conditions with the APO in TM and without the APO were compared. In this study, participants walking with the APO in TM did not decrease the self-selected walking speed (change of 0.01 ± 0.02 m/s) and did not change the spatiotemporal parameters or lower-limb kinematics compared to walking without the APO. Thus, study results suggested that the penalty of wearing the device (weight of about 5 kg) did not negatively affect study participants’ gait performance and that the APO’s mechanical structure is minimally restrictive and allows good kinematic coupling with the user.

Concerning the hip exoskeleton assistive strategies, in most cases, hip assistance was set as biomimetic torque (i.e., extension-flexion) vs. gait phase profile^16,18^. A recent study involving stroke survivors^22^investigated the effect of bilateral and unilateral biomimetic hip assistance and showed that, on average, bilateral hip assistance increased gait speed more (17.6%) than unilateral assistance (11.1%) relative to the baseline; consistently with other studies suggesting that unilateral hip assistance may be suboptimal^36^, given the relevant role of the inter-limb coordination during walking^37^. Notably, in the study investigating unilateral and bilateral hip assistances^22^, the assistance strategy that maximized benefits varied among participants, suggesting that individualizing gait assistance per user could contribute to benefit, consistent with previous studies with healthy subject^38,39^or targeting the ankle joint^40,41^.

In our previous study, we showed that after two days of gait training with the APO controlled with the proposed assistive strategy, 14 individuals with acquired brain injury significantly increased the overground walking distance in the 6MWT by 23 ± 3% in the NoAPO condition post training compared to pre training. Noteworthy, in the above cited study^20^, the effect of walking with the APO in AM consisted of a reduction of the walking speed compared to walking without the device within the same session. This difference among studies might be explained by three main technical improvements achieved in the current version of the APO, namely, (i) the APO version used in this work has a lower weight (about − 1 kg), (ii) the middle-level control is based on a more accurate gait phase estimation algorithm, and therefore, the torque vs. phase profile is more repeatable^36^ and (iii) on average the torque amplitude was set higher.

This study demonstrates that powered and bilateral hip exoskeletons may have a positive impact on the mobility of individuals in the chronic post-stroke phase and that they may hold potential for establishing a new standard of care and provision of assistance for this population. However, the small sample size (*n* = 6) limits the generalizability and the relevance of the study results. Future clinical studies should assess the effectiveness of the proposed method on a larger sample and a broader population of individuals with neurological disorders including subjects in the subacute phase, with different levels of functional mobility, and different levels of spasticity. To date, a mechanistic explanation of the observed improvements in gait speed is still lacking. Thus, to get a deeper insight into the observed improvement, muscular activities, muscular synergies, and corticomotor excitability elicited by the hip assistance should be evaluated. By investigating these physiological and neuromuscular aspects, researchers can better understand how powered exoskeleton facilitate improvements in mobility and inform the development of more targeted and effective interventions for individuals with neurological disorders.

In addition, to promote the use of a hip exoskeleton as an assistive tool for locomotor training or for extending mobility in a real-world scenario, research teams should prioritize enhancing usability and ease of use of these devices. Further advances are needed to ensure that hip exoskeletons are safe, reliable, and practical for everyday use. This include addressing factors such as comfort, portability, battery durability and user interface design for intuitive operation. Finally, attention should be directed to customized fitting to accommodate a diverse range of body anthropometries and gait patterns.

## Methods

### Powered bilateral hip exoskeleton and assistive strategy

The Active Pelvis Orthosis (APO) is a bilateral powered robotic hip orthosis (or exoskeleton) designed to power hip flexion or extension gait movements by providing smooth assistive torques at the hip, adapting to natural gait variations. The APO can provide support to individuals who retain walking capacity with or without assistive tools and present mild-to-moderate gait impairments. Since the APO provides neither weight support nor balance assistance, it is not intended for individuals who require assistance or body weight support to walk. The APO system used for this study was based on the same mechatronic architecture as previously reported prototypes^25,42^, with additional design optimizations for portability and weight reduction to 5.7 kg.

From the mechatronics viewpoint, the APO is built around an aluminum frame that surrounds the user’s hips and posterior pelvis, and interfaces with the trunk and hips via customisable orthotic cuffs (one for the lumbar part with shoulder straps and one for the thigh parts). The APO frame carries a backpack –housing the control electronics and battery– and two actuation units, one on each side, employing a series-elastic actuator architecture^43^. Each actuation unit is deployed along two parallel axes. The first axis (posteriorly located) is the output shaft of a 70-Watt-BLDC motor coupled with a 1:100 harmonic drive and a torsional spring (stiffness equal to 220 N∙m/rad), ensuring compliant interaction with the lower-limb segment. The torsional spring deformation is measured by a 17-bit absolute magnetic encoder. The second axis (anteriorly located) is collocated with the hip flexion–extension axis and is featured with a 13-bit absolute magnetic encoder for hip angle measurement. The two parallel axes are connected by a 4-bar linkage mechanism. For each side, the transfer of assistive torque from the actuation unit to the hip articulation is provided by a thigh cuff connected to the actuation output axis through a rigid carbon-fiber link. Each actuation unit can deliver a peak torque of 16 N·m over a range of movement between − 30 and 110 deg and is featured with a non-collocated passive degree of freedom allowing the user to freely perform hip ab/adduction movements.

The APO can deliver the desired torque pattern through a hierarchical control algorithm relying on accurate gait phase recognition for synchronization of the assistive action with the intended movement of the user. The APO control system runs on a real-time controller (NI SbRIO9651 processor, National Instruments, Austin, Texas, US) featured with both a dual-core ARM controller and a Field-Programmable Gate Array (FPGA) processor. A high-level control layer (running at 100 Hz on the ARM processor) generates a desired phase-locked torque profile following a precise gait-phase estimation, which is obtained by continuously tracking the hip joint angles through a pool of Adaptive Oscillators and using a wavelet-based initial contact detection method for the cyclic smooth reset of the gait phase at each stride^44^. The desired torque is computed for each hip joint, as the sum of two Gaussian functions (each Gaussian function can be used to assist gait either during hip flexion or hip extension)^45^. For each function, the experimenter can tune the following parameters: phase (% of the gait phase where the peak occurs), amplitude (N·m) of the torque peak (positive/negative to deliver flexion/extension assistance respectively), and duration of the assistance (% of the gait phase). The duration is the width of the Gaussian function. A closed-loop torque compensator running on the FPGA at 1 kHz is responsible for tracking the desired assistive torque to be delivered to the user.

The APO can be controlled in two different operational modes, namely transparent mode (TM) and assistive mode (AM). In TM, the high-level controller sets the desired torque to 0 N·m and the APO is transparent to the user, i.e., the user can walk, and the APO provides minimal-to-null resistance to the user. In AM, the APO provides the phase-locked desired torque.

In this study, the desired assistive torque profile was designed according to a method used in our prior clinical invetigation^20,26^(patent application nr. WO2022137031)^26^. According to this methodology, a different set of phase timing, amplitude, and duration of each Gaussian function was selected based on volunteers’ gait impairments. In this study, two different assistive profiles resulted on the paretic side based on the presence of knee hyperextension deficit during the stance phase. For those volunteers who exhibited knee hyperextension, we opted for a double flexion torque-to-phase assistive profile, i.e., an assistive profile with two positive Gaussian curves, the first during the stance phase (synchronously with the knee hyperextension), and the second during the swing phase. For those volunteers who did not exhibit knee hyperextension, we opted for an extension-flexion assistive torque-to-phase profile, i.e., an assistive profile mimicking the biological hip torque, with a negative Gaussian curve during the stance phase and a positive Gaussian curve during the swing phase. Whereas, on the nonparetic side, for all study participants, the assistive profile mimicked the biological hip flexion–extension torque with the aim to reduce the necessary effort to compensate for impairments of the paretic side.

## Experimental protocol

Individuals exhibiting mild-to-moderate gait impairments, due to a stroke, were enrolled in this study. Participant inclusion criteria included the following: (1) age in the range [18, 80] years old, (2) time since stroke event > 3 months, (3) completion of the basic rehabilitation program, (4) capability of independent gait, even with assistive tools (i.e., Functional Ambulation Classification ≥ 4; Medical Research Council muscle strength score in the range (2,4]), (5) ability to walk on a treadmill, even at low speeds, (6) Pelvis width in the range [34, 41] cm. Throughout the study duration, participants were allowed to use their assistive tools or bracing, such as cane, crutches, and ankle foot orthosis. Exclusion criteria included: (1) cognitive impairment (Mini-Mental State Examination score < 21), (2) severe anxiety or depression (State-Trait Anxiety Inventory-Y > 44, Beck Depression Inventory-II > 19), (3) relevant comorbidities (e.g., chronic heart failure, uncontrolled diabetes or hypertension, chronic obstructive pulmonary disease, severe hip/knee osteoarthritis, severe osteoporosis, severe sensory deficit), (4) implantable cardiac devices, such as pacemakers or automatic defibrillators and (5) physician disapproval. We enrolled six participants (1 female; age: range 24–76 years; time since stroke event: range 16–120 months; 4 left and 2 right-side hemiparesis). An exhaustive description of all study participants is reported in Supplementary Table I. All participants were informed about the purpose of the study, procedures, and data treatment and signed an informed consent before starting experimentation.

**Fig. 5:**
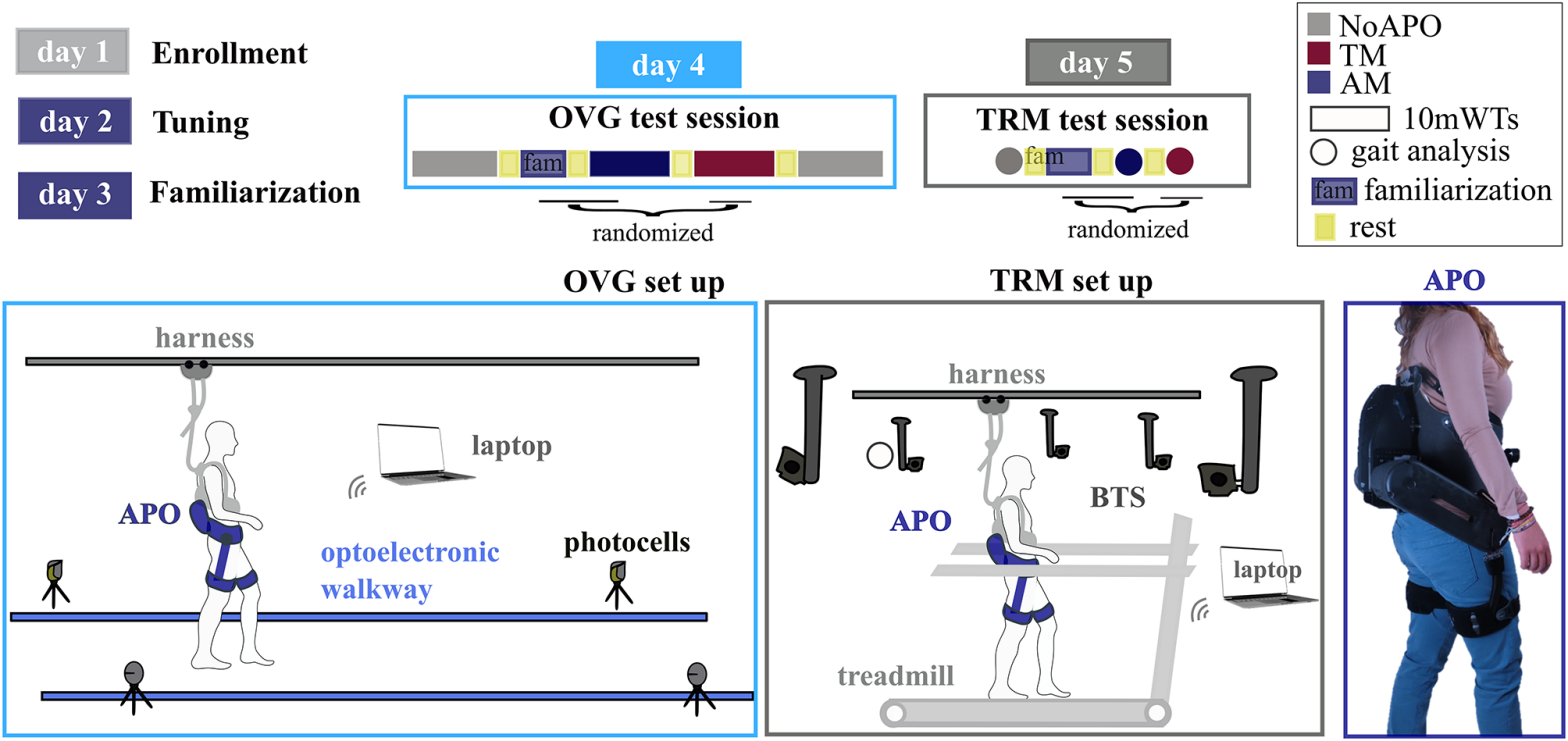
Schematic of the experimental protocol and setup. The experimental protocol consisted of 5 sessions. The first session (*Enrollment*) included the evaluation of the study participants’ eligibility and a gait analysis overground without the APO. The second (*Tuning*) and the third (*Familiarization*) session were devoted to the tuning of the APO assistive profile and the familiarization with the exoskeleton assistance. Following these sessions, participants performed two Test sessions: one overground (OVG, day 4) and one on the treadmill (TRM, day 5). The OVG test session included 10mWTs under three different walking conditions: (i) without the APO at the beginning and at the end of the session (NoAPO_baseline_, NoAPO_final_, grey), (ii) with the APO in transparent mode (TM, bordeaux), (iii) with the APO in assistive mode (AM, blue). During the TRM test session, participants gait performance under the same three different conditions (NoAPO, TM, AM, circle) was assessed through gait analysis tests. On the right, a representative picture of the APO.

The study was carried out at the clinical center IRCCS Fondazione Don Carlo Gnocchi (Florence, Italy). The experimental protocol was approved by the local Ethics Committee, namely the Comitato Area Vasta Centro Toscana (Protocol ID: CLs + + 2ndCS; approval number: 16454_spe) and notified to the Italian Ministry of Health. Experimental procedures were in accordance with the Declaration of Helsinki. For each participant, the protocol included 5 sessions conducted on 5 different days over 2 weeks (Fig. 5).

In the first *Enrollment*session, clinicians verified whether the recruited patient complied with inclusion/exclusion criteria. In case the participant was successfully enrolled, the participant’s gait was assessed through overground gait analysis (GA). For the GA test, the subject was asked to walk at the self-selected comfortable gait speed at least 10 times along a 10-meter walkway in a gait analysis laboratory instrumented with an optoelectronic motion capture system (Smart-DX BTS Bioengineering, Milano, Italy) and force platforms (P6000, BTS Bioengineering, Italy). Twenty-two reflective optical markers were placed on anatomical landmarks, following the Davis protocol^46^. Based on the kinematic and kinetic gait profiles, the clinicians identified the patient’s gait impairments.

The second *Tuning*session was devoted to setting the hip assistance profiles delivered by the bilateral hip exoskeleton according to the procedure adopted in a previous study^20,]^ and briefly recapped in the previous section. First, after helping the volunteer to wear the APO and adjusting the robot’s settings to comply with the user’s anthropometric sizes, each recruited subject was instructed to walk at the self-selected speed with the APO in TM back and forth along a 23-meter walkway to record his/her hip kinematics through the exoskeleton onboard sensory system. Second, by relying on data from GA and APO onboard sensory system, experimenters designed the hip assistive torque-to-phase profile. In order to fine tune the identified initial set of parameters, each volunteer was then instructed to walk multiple times along the walkway: torque level was gradually increased to reach a maximum value close to BM/10 Nm/kg (BM = body mass), while ensuring a comfortable human-robot interaction, based on volunteer’s feedback.

During the third *Familiarization* session, participants freely selected their walking speed to learn how to interact with assistance. The familiarization consisted of about 40 min of non-continuous overground walking and about 5 min of treadmill walking in assistive mode.

After the *Tuning* and the *Familiarization* sessions, the participants underwent two *Test* sessions, one overground and one on the treadmill. Each *Test* session was devoted to comparing walking performance under three different walking conditions: (i) walking without the APO, (ii) walking with the APO in TM, (iii) walking with the APO in AM.

In the *Overground Test* session, the participant’s gait was assessed through 10mWTs performed at the self-selected comfortable walking speed. For each walking condition, four consecutive 10mWTs were performed. For the 10mWTs study participants were instructed by a physical therapist to walk along the 23-meter walkway at their self-selected comfortable walking speed. The gait speed was measured over a 10-meter segment roughly placed in the mid of the 23-m walkway (i.e., the initial 8 m were used to allow the volunteer to reach the steady-state walking speed and the exoskeleton’s middle level control to synchronize with the user gait; the last 5 m were left to allow the volunteer to decelerate for stop and the robot to stop the assistance). Each session started with four 10mWTs without the APO (NoAPO_baseline_) to measure the baseline gait speed. Next, four 10mWTs were performed with the APO in TM and AM, and at the end of the session, additional four 10mWTs without the APO (NoAPO_final_) were recorded.

During the *Treadmill Test* session, the participant’s kinematics was assessed through a GA during treadmill-based walking at their preferred walking speed. The preferred walking speed in this session was selected with the APO in AM. For each walking condition, participants walked two minutes on the treadmill at the self-selected speed, the last minute was recorded by the motion capture system. In the walking tasks with the APO, the markers masked by the exoskeleton (i.e., greater trochanters and sacrum) were attached to the structure of APO in correspondence with the anatomical landmarks.

In both the *Test* sessions (overground, treadmill), the sequential order of the APO conditions (i.e., AM, TM) was randomized across participants to avoid order bias, due to fatigue or learning effects. In addition, before the tests in AM, a short familiarization was performed to refresh the gait pattern mediated by the assistance (i.e., approximately 10 corridors overground and 3 min on the treadmill). Between different walking conditions, a short rest was performed according to the subject’s needs.

## Outcomes and data analysis

The primary outcome of the study was the overground walking speed with vs. without the APO. Secondary outcomes were the spatiotemporal parameters during overground walking and lower-limb kinematics assessed during treadmill walking with vs. without the APO.

The overground walking speed was measured by means of two photocells (Witty, Microgate S.r.l., Italy) placed 10 m from each other, roughly in the mid of the 23-m walkway. Study participants performed the corridor four times per each walking condition. Across corridors the four gait speeds measured under the same condition were aggregated and reported as median. Difference in gait speed were compared with the small (0.06 m/s). and substantial MCID (0.10 m/s).

The APO data (including hip angle and torque) were acquired and analysed offline in Matlab (Mathworks Inc, Natick, USA). From the hip angle, we estimated the hip joint velocity and the actual delivered assistive power. Hip angle, torque, and power signals were then segmented into single strides using the initial foot contact estimated online by the method reported in.

The gait spatiotemporal parameters (e.g., cadence, stride time and step length) were measured in the middle portion of the 10-meter segment using an Optoelectronic walkway (Optogait, Microgate, Bolzano, Italy).

Gait biomechanics was measured using a stereophotogrammetric motion capture system (Smart-DX, BTS Bioengineering, Milano, Italy). Twenty-two reflective optical markers were placed on anatomical landmarks, following the Davis protocol. In the case of gait analysis with the APO, the three following markers were masked by the APO and were moved from the anatomical landmark to the robotic frame: the marker on the sacrum, and the markers on the left and right greater trochanter. Trajectories of optical markers were collected and processed through the BTS Smart software (Smart-DX, BTS Bioengineering, Milano, Italy). Mean and standard deviation values of the lower-limb kinematic profiles were computed. Range of Motion (RoM) on the sagittal plane of hip, knee and ankle joints was computed as difference between maximum flexion and extension joint angle.

All collected data were analysed by means of custom Matlab routines (MathWorks, Inc., Natick, MA, USA).

### Statistical analysis

The normality of the data distribution was checked by the Shapiro-Wilk test (α = 0.05). All measures were reported with descriptive statistics as mean and standard errors or as median and (25th, 75th) percentiles when the normality assumption was violated.

To infer statistical differences among walking conditions (NoAPO, AM, TM), firstly, the one-way repeated-measures ANOVA statistical test was conducted (α = 0.05) since the normality test failed to reject the null hypothesis for the measures of gait speed, spatiotemporal and kinematics parameters. Secondly, after observing significant differences, a post-hoc analysis was performed using the Fischer’s Least Significant Difference correction. When the sphericity assumption did not hold using the Mauchly’s test, the Epsilon (ε) correction was used [Greenhouse-Geisser correction (ε>0.75) or Huynh-Feldt correction (ε<=0.75), depending on the ε value]. Additionally, a paired t-test was performed to statistically analysis the differences in the energy provided by the APO to the paretic and non-paretic sides across participants.

## Electronic supplementary material

Below is the link to the electronic supplementary material.

Supplementary Material 1

## Data Availability

The datasets generated and/or analysed during the current study are available from the corresponding author on reasonable request.
