# Supplementary material for: Bilateral hip exoskeleton assistance enables faster walking in individuals with chronic stroke-related gait impairments
Source: Sci Rep. 2025 Jan 15;15:2017. doi: 10.1038/s41598-025-86343-x (PMC11735669; doi:10.1038/s41598-025-86343-x)
Supplement: Supplementary file 1 — Supplementary Material 1 [file 41598_2025_86343_MOESM1_ESM.docx]

# Bilateral hip exoskeleton assistance enables faster walking in individuals with chronic stroke-related gait impairments

**Chiara Livolsi^1,2,*^, Roberto Conti^3^, Tommaso Ciapetti^4^, Eleonora Guanziroli^5^, Þór Friðriksson^6^, Ásgeir Alexandersson^6^, Emilio Trigili^1,2^, Francesco Giovacchini^3^, Raffaele Molino Lova^4^, Alberto Esquenazi^7^, Franco Molteni^5^, Simona Crea^1,2,4,§,^ & Nicola Vitiello^1,2,4,§^.**

**^1^** The BioRobotics Institute, Scuola Superiore Sant’Anna, Pontedera, Pisa, Italy

**^2^** Department of Excellence in Robotics & AI, Scuola Superiore Sant’Anna, Pisa, Italy

**^3^** IUVO S.r.l., Pontedera, Pisa, Italy

**^4^** IRCCS Fondazione Don Carlo Gnocchi ONLUS, Florence, Italy

**^5^** Villa Beretta Rehabilitation Center, Valduce Hospital, Costa Masnaga, Lecco, Italy

**^6^** Össur, Reykjavík, Iceland

**^7^** Department of PM&R, MossRehab and Einstein Healthcare Network, Elkins Park, PA, USA

**^*^** Corresponding author: [chiara.livolsi@santannapisa.it](mailto:chiara.livolsi@santannapisa.it)

**^§^** SC and NV share the senior authorship.

## Supplementary information

Table I. **Study participants’ characteristics**

| Participant ID# | ID1 | ID2 | ID3 | ID4 | ID5 | ID6 |
| --- | --- | --- | --- | --- | --- | --- |
| Age (y) | 58 | 49 | 76 | 57 | 24 | 69 |
| Sex | male | male | male | male | female | male |
| Height (cm) | 175 | 175 | 170 | 170 | 173 | 177 |
| Weight (kg) | 90 | 85 | 80 | 76 | 69 | 85 |
| Cause of stroke | ischemic | hemorrhagic | ischemic | hemorrhagic | ischemic | hemorrhagic |
| Side of paresis | right | right | left | left | left | left |
| Chronicity (months) | 60 | 120 | 46 | 35 | 16 | 18 |
| Gait impairment identified during stance | Knee hyperextension | Deficit in hip extension,  knee buckling, deficit in plantarflexion | Knee buckling, deficit in plantarflexion | Knee hyperextension, deficit in plantar flexion | Knee hyperextension, deficit in plantar flexion | Deficit in hip extension, deficit in plantarflexion |
| Gait impairment identified during swing | Deficit in hip flexion, stiff knee, reduced ground clearance | Deficit in hip flexion, stiff knee, reduced ground clearance | Deficit in knee flexion, reduced ground clearance | Deficit in hip flexion, stiff knee, reduced ground clearance | hip hiking, stiff knee, reduced ground clearance | Deficit in knee flexion, reduced ground clearance |
| Regular assistive tool | none | cane + AFO | none | none | AFO | crutch |
| Baseline walking speed (m/s) | 0.7 | 0.9 | 0.8 | 0.9 | 1.2 | 0.4 |

Table II. **Step length and stance period for all study participants during overground walking tests with and without the APO**. Spatial and temporal gait parameters of the paretic and nonparetic side are indicated for the following experimental conditions:i) without the APO at the beginning of the session (NoAPObaseline), (ii) with the APO in assistive mode (APO AM), (iii) with the APO in transparent mode (APO TM), iv) without the APO at the end of the session (NoAPOfinal).

|  | **Paretic side** | | | | **Nonparetic side** | | | |
| --- | --- | --- | --- | --- | --- | --- | --- | --- |
|  | **NoAPO baseline** | **APO AM** | **APO TM** | **NoAPO final** | **NoAPO baseline** | **APO AM** | **APO TM** | **NoAPO final** |
| **Step length (cm)**  **Mean (std)** | 41.75 | 52.13 | 47.31 | 49.00 | 42.50 | 49.06 | 44.31 | 48.38 |
|  | (3.49) | (1.49) | (0.95) | (1.73) | (1.66) | (1.62) | (1.86) | (2.72) |
|  | 68.88 | 69.44 | 66.44 | 67.75 | 65.25 | 65.63 | 62.94 | 67.00 |
|  | (1.14) | (1.98) | (0.86) | (1.79) | (0.83) | (1.55) | (0.88) | (0.71) |
|  | 54.00 | 61.56 | 57.38 | 59.50 | 56.63 | 59.00 | 57.13 | 59.88 |
|  | (1.37) | (3.16) | (1.95) | (1.12) | (1.08) | (3.21) | (1.44) | (1.14) |
|  | 61.00 | 65.13 | 59.50 | 62.38 | 49.25 | 52.38 | 49.75 | 49.13 |
|  | (2.03) | (1.29) | (1.26) | (1.67) | (0.83) | (0.65) | (1.29) | (0.74) |
|  | 76.25 | 77.88 | 79.38 | 84.63 | 64.75 | 65.00 | 61.81 | 64.50 |
|  | (1.30) | (2.51) | (1.64) | (2.58) | (0.83) | (2.55) | (2.70) | (0.87) |
|  | 44.13 | 51.38 | 44.38 | 46.50 | 33.75 | 46.63 | 36.75 | 36.25 |
|  | (2.16) | (0.74) | (1.50) | (0.79) | (1.30) | (1.19) | (1.17) | (3.21) |
| **Stance time (%gait cycle)**  **Mean (std)** | 58.93 | 56.74 | 57.58 | 57.65 | 69.40 | 66.89 | 68.56 | 65.89 |
|  | (1.42) | (0.98) | (0.65) | (1.92) | (1.94) | (2.18) | (1.59) | (2.10) |
|  | 60.48 | 61.18 | 61.84 | 60.90 | 65.06 | 65.32 | 66.75 | 64.73 |
|  | (1.57) | (0.72) | (0.84) | (2.13) | (0.88) | (0.64) | (0.72) | (1.03) |
|  | 69.19 | 64.63 | 66.20 | 57.26 | 64.69 | 66.72 | 66.94 | 60.70 |
|  | (2.69) | (1.90) | (3.30) | (3.07) | (3.88) | (2.16) | (3.21) | (2.93) |
|  | 52.55 | 54.60 | 53.58 | 52.98 | 64.19 | 63.03 | 65.40 | 64.93 |
|  | (0.46) | (0.59) | (0.63) | (0.54) | (0.93) | (0.43) | (0.22) | (1.03) |
|  | 57.56 | 59.81 | 58.96 | 56.89 | 67.28 | 67.73 | 68.41 | 66.64 |
|  | (2.41) | (1.35) | (1.70) | (1.63) | (0.99) | (1.23) | (0.91) | (0.92) |
|  | 60.84 | 58.73 | 61.79 | 60.88 | 70.80 | 64.99 | 68.99 | 67.28 |
|  | (4.42) | (2.23) | (4.56) | (2.12) | (0.87) | (1.69) | (1.42) | (0.51) |


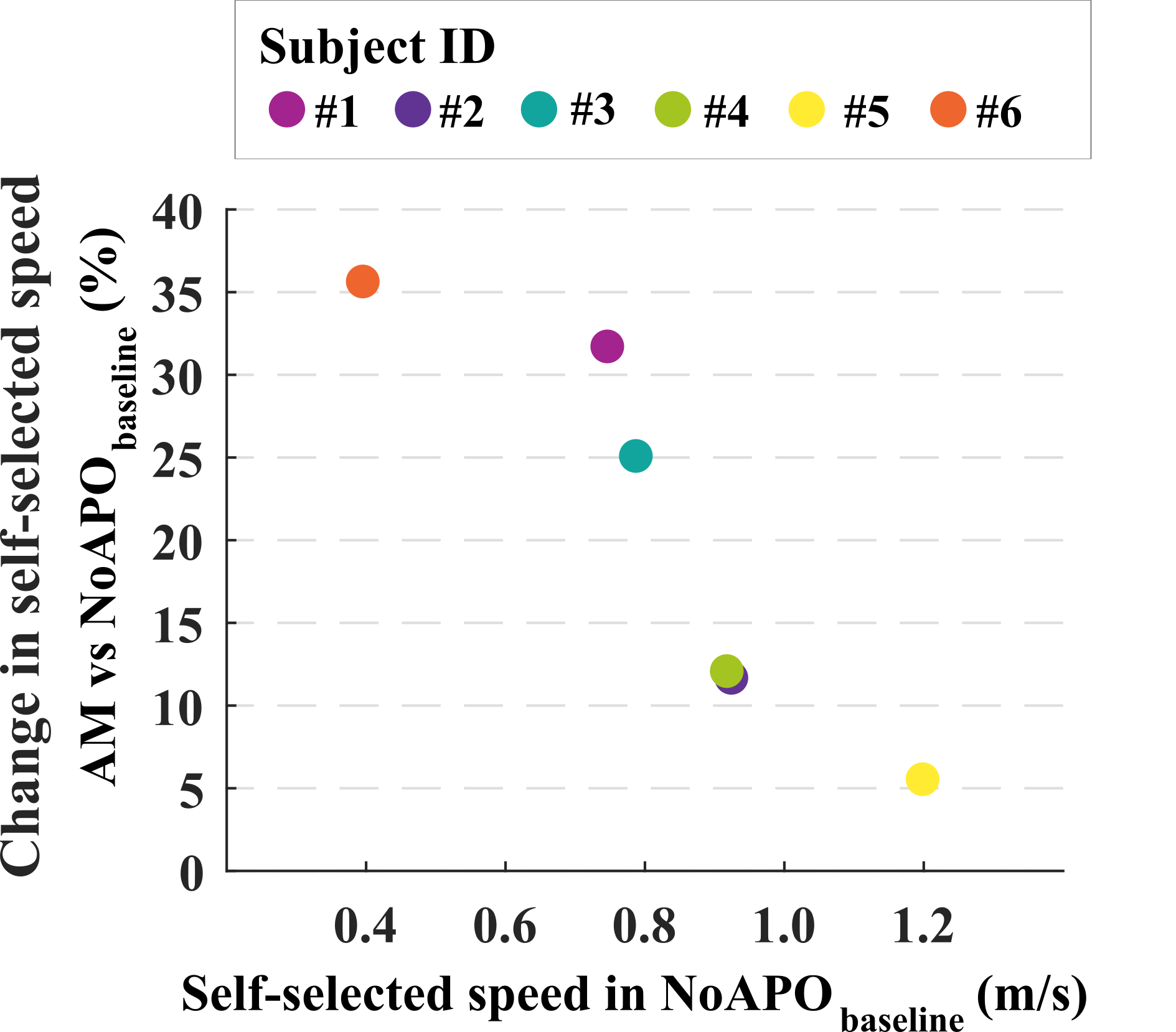


Supplementary Figure 1 Percentage change in the self-selected speed with the APO in AM versus the baseline gait speed without the APO for each study participant.
